# Perception, Cognition, and Action in Hyperspaces: Implications on Brain Plasticity, Learning, and Cognition

**DOI:** 10.3389/fpsyg.2019.03000

**Published:** 2020-01-22

**Authors:** Haluk Ogmen, Kazuhisa Shibata, Arash Yazdanbakhsh

**Affiliations:** ^1^Laboratory of Perceptual and Cognitive Dynamics, Electrical & Computer Engineering, Ritchie School of Engineering & Computer Science, University of Denver, Denver, CO, United States; ^2^Laboratory for Human Cognition and Learning, RIKEN Center for Brain Science, Wako, Japan; ^3^Department of Psychological and Brain Sciences, Computational Neuroscience and Vision Lab, Center for Systems Neuroscience, Boston University, Boston, MA, United States

**Keywords:** hypercube, brain plasticity, sensorimotor, four-dimensional, neural representations, learning, brain development

## Abstract

We live in a three-dimensional (3D) spatial world; however, our retinas receive a pair of 2D projections of the 3D environment. By using multiple cues, such as disparity, motion parallax, perspective, our brains can construct 3D representations of the world from the 2D projections on our retinas. These 3D representations underlie our 3D perceptions of the world and are mapped into our motor systems to generate accurate sensorimotor behaviors. Three-dimensional perceptual and sensorimotor capabilities emerge during development: the physiology of the growing baby changes hence necessitating an ongoing re-adaptation of the mapping between 3D sensory representations and the motor coordinates. This adaptation continues in adulthood and is quite general to successfully deal with joint-space changes (longer arms due to growth), skull and eye size changes (and still being able of accurate eye movements), etc. A fundamental question is whether our brains are inherently limited to 3D representations of the environment because we are living in a 3D world, or alternatively, our brains may have the inherent capability and plasticity of representing arbitrary dimensions; however, 3D representations emerge from the fact that our development and learning take place in a 3D world. Here, we review research related to inherent capabilities and limitations of brain plasticity in terms of its spatial representations and discuss whether with appropriate training, humans can build perceptual and sensorimotor representations of spatial 4D environments, and how the presence or lack of ability of a solid and direct 4D representation can reveal underlying neural representations of space.

## Introduction

Species operate in space and their behavioral success depends on how well they process, represent, store, and recall spatial information. In addition to being a fundamental aspect of sensorimotor behavior, the concept of space plays an important role in our understanding of higher cognitive functions. Indeed, the concept of space has been central to the thinking of many philosophers such as Plato, AlHazen, Descartes, Hume, and Kant to name a few (Huggett, 1999). Questions such as whether space is *a priori* or learned, whether it is objective or subjective have been debated. The concepts of space and time and their relationship shape theories in modern physics. Given this background, it goes without saying that the concept of space also plays a major role in psychology and neuroscience. For example, based on children’s spatial reorientation and navigation behavior, Spelke et al. (2011) concluded that two systems of human core knowledge (i.e., ancient, innate, and universal systems) reflect the primary *properties of Euclidian plane geometry*. She suggested that these systems lack the power of abstract generalization; however, language and symbolic representations allow children to combine productively their core knowledge to attain abstract thoughts that form the foundations of abstract mathematical geometry (Spelke et al., 2011). According to the Piagetian theory, higher cognitive functions are built upon sensorimotor schema, which represent the operation of sensory and motor systems in space and time (Piaget, 1936, 1950). The progression from sensorimotor stage to formal operational stage takes place as ego-centric “subjective” sensorimotor representations, i.e., representations whose reference frames are based on the subject, such as head-centered representations, become coordinated across initially independent sensory spaces (e.g., ego-centric visual space, ego-centric tactile space) to give rise to exo-centric (i.e., reference-frames) based outside of the subject “objective” representations (e.g., object-centered representations) (Piaget, 1936, 1950). Neurophysiological studies revealed multiple representations of space in various brain areas, including ego-centric (Gross and Graziano, 1995; Grieves and Jeffery, 2017) and exo-centric representations (Olson, 2003). Notwithstanding specific theoretical stances and underlying neurophysiological correlates, these approaches focus on three-dimensional (3D) or two-dimensional (2D) representations simply because our environment is 3D and its projections are 2D. Our visual system receives on our retinas a pair of 2D projections of a 3D environment. By using multiple cues, such as disparity, motion parallax, perspective, and shading, our brains can construct 3D representations of the world from its 2D projections on our retinas.

**FIGURE 1:**
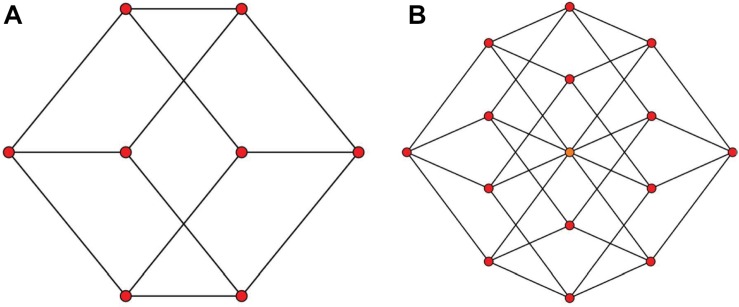
Two-dimensional projections of a 3D and a 4D object. It is easy and accessible for us to have a solid 3D impression of **(A)** as a cube, however, looking at **(B)** doesn’t pop a solid 4D impression of a hypercube. Panels **(A,B)** are from: http://en.m.wikipedia.org/wiki/File:3-cube column graph.svg and http://commonswikimedia.org/wiki/File:4-cube column graph.svg, respectively, and have been released into the public domain by their author Geoff Richards (Qef).

A natural question that arises is, if our brains can construct 3D representations from the 2D projections (Figure 1A), can it also construct 4D spatial representations from 2D projections that represent a spatially 4D world (Figure 1B)? This question touches a fundamental theme regarding the limits of plasticity in the brain: Are our brains hard-wired to be limited to 3D representations because it evolved and developed in 3D worlds, or do they have inherent plasticity to represent arbitrary dimensions? The neurophysiology and neuroanatomy of the brain indicate high-dimensional internal representations: For example, the representation for an object not only includes three-dimensional space but also other feature dimensions like brightness, hue, texture, curvature, etc. However, whether these internal high-dimensional representations can be translated to *higher dimensions for the external space* remains unanswered. Support for brain plasticity in spatial representations comes from inherent plasticity that allows to cope for natural changes that occur, in particular, during the developmental period where three-dimensional perceptual and sensorimotor capabilities emerge. A fundamental aspect of development is that the physiology of the growing baby changes hence necessitating an ongoing re-adaptation of the 3D representations and the mapping between 3D sensory representations and the motor coordinates. This adaptation continues in adulthood. But is this adaptation general to deal with drastic changes such as going from 3D to 4D? Hence, a fundamental question is whether our brains are inherently limited to 3D representations of the environment because we are living in a 3D world. Alternatively, our brains may have the inherent capability of representing arbitrary dimensions; however, 3D representations emerge from the fact that our development and learning take place in a 3D world.

## Operationalization of the Concept

Studies exploring 4D environments focused on different aspects of the underlying concept. For example, the titles of these studies refer to the phenomenon under study as “*4D spatial reasoning*,” “*4D spatial intuition*,” “*4D spatial judgments*.” “*4D spatial representations*.” They also use different variables to operationalize the concept. Such a diversity is expected because our spatial competencies are multi-faceted and have different neural correlates. We can *perceive* three-dimensional objects, we can *reason* about three-dimensional space and objects in abstract ways, and we can *navigate* in the 3D space. Although these abilities do have some communalities, their underlying computations and neural correlates are not identical. For example, when we state that we perceive a 3D object, we may be referring to the perceptions that arise from a 2D drawing with perspective cues versus those that arise in a virtual reality headset. Although in both cases we “perceive a 3D structure,” the phenomenal experience from a 2D drawing vs. from virtual reality are extremely different suggesting differences in their neural representations.

## Summary of Findings

Aflalo and Graziano (2008) studied subjects’ path-integration ability in 4D environments to assess their “four-dimensional spatial reasoning.” Subjects (five adults) were asked to navigate in a 4D maze and, upon reaching the end of the maze, to point to the location of the starting point of the maze. The angular difference between subjects’ response and the true direction quantified the error in path integration. The 4D maze was constructed by mapping the 4D stimulus on a 2D display through projective geometry (this is called the “*projection technique*” of 4D stimulus generation). An additional cue was added for the fourth dimension: The extremes of the fourth dimension’s axis (±∞)were labeled “hot” and “cold” and hues between red and blue were used to signal to the subject where s/he is along the fourth dimension’s axis. Subjects were allowed to practice on their own computer at home and spent 15 min to 1 h per day for several weeks. They were given feedback after each trial. During the initial trials in laboratory testing for the 4D maze, subjects’ performance improved rapidly (ca. 20 trials; average trial duration ranged from 45 s to 2.7 min) and remained thereafter at a plateau level. A second improvement in performance was observed after the plateau phase (ca. 160 trials). Aflalo and Graziano (2008) compared their subjects’ performance to those of two theoretical (simulated) models that were designed to solve the puzzle by using *only* 3D spatial reasoning. With subjects performing better than these models, Aflalo and Graziano (2008) concluded that subjects were capable of 4D spatial reasoning, as assessed by the path-integration task.

Ambinder et al. (2009) used 4D tetrahedrons and presented subjects successive slices of the stimuli as it would happen if the subject were to walk through the fourth dimension’s axis (this is called the “*slicing technique*” of 4D stimulus generation). Observers (four adults) judged the distance between two vertices or the orientation of a surface plane. No feedback was given. They found a significant positive correlation between reported distances/orientations and the true 4D distances/orientations (among conditions and subjects, *R*^2^ values ranged from 0.29 to 0.81). They concluded that humans can represent and make geometrical judgments (distance and orientation) about 4D objects.

In a follow-up study, Wang (2014a) noted that the path-integration task used by Aflalo and Graziano (2008) can be solved by using exclusively 3D information. There is also evidence that navigation tasks may be based on graphs rather than geometrical maps (Chrastil and Warren, 2014). Wang (2014a) also pointed out that length and orientation judgments used by Ambinder et al. (2009) are 1D and 2D geometric properties, respectively. To eliminate these lower dimensional solutions, she selected a 4D property, the hyper-volume of 4D objects, as the dependent variable. To present the 4D stimuli, she used a “*rotation technique*,” where the 4D object is rotated around a selected plane. No feedback was given to the subjects (three adults). Results showed significant correlations between reported and actual hypervolumes (ß values ranged from 0.44 to 0.73) and Wang concluded that humans can have 4D representations that provide estimates of inherently 4D properties, such as hyper-volume.

Miwa et al. (2018) designed an interactive environment wherein a 4D hypercube was projected to a 3D space by using perspective projection. A hypercube was centered at the origin of the 4D space and each of the eight sides of the 4D cube was displayed with a different color. Perspective projection makes parallel lines converge at infinity^1^ and this point of convergence is called the “vanishing point.” When the parallel lines are also parallel to one of the axes of the coordinate system, these points are called “*principal vanishing points*.” These principal vanishing points were displayed along with the stimulus. The observer could change her point of view of the stimulus by manipulating principal vanishing points. This allowed the observer to actively visualize and explore the stimulus. Before the start of the main experiment, observers were given up to 3 h to actively visualize and explore the stimulus. They were then tested on their ability to navigate along checkpoints in the 4D space. These checkpoints were chosen randomly with the constraint that three or four sides of the hypercube are simultaneously visible from the checkpoint. Of their 12 subjects (mean age: 20.5 years), one dropped out, three had poor performance (measured by the proportion of correctly visited checkpoints) and the remaining eight had successful performance (≥70%). In a second experiment, they used the so-called “color cube test,” where an *N*-dimensional cube is constructed with each side having a different color. This cube is then projected to a *N* – 1 dimensional space and observers are shown *N* different views of the cube in the *N* – 1 dimensional space. The task of the observer was to answer questions like “*what color is the side of the cube which is positioned opposite to the green side?*” To accomplish this task in 3D, observers typically mentally rotate a 3D mental representation of the cube to understand the relationships between different sides/colors. Of the twelve subjects, two dropped out, three had poor performance whereas the remaining seven had good performance (≥70%). Based on these results, the authors concluded that “humans are capable of learning 4D representations.”

While higher order visual areas are involved in 3D vision (Tsao et al., 2003), neural responses in early visual areas are modulated by stimulus 3D configuration (Dobbins et al., 1998). Qian and Yazdanbakhsh (2015), suggested a neural model for interactions between higher and lower visual areas encoding distance and size that can be one of bases of size constancy – perceiving an object size irrespective of its distance, as one of the lower order spatial recognition processes. As such, if human direct 4D percept materializes, we may reach to broader forms of visual object constancy that signify higher order scene groupings to be reflected by modern artists and virtual reality developers.

## Outstanding Problems: Operationalization of the Concept

As mentioned previously, human perception, cognition, and action are multi-faceted. When we study human perception, cognition, and action in 3D, we introduce numerous experimental paradigms and variables intended to capture different aspects of brain processes and behaviors. Similarly, studies of 4D used different approaches such as navigation performance and a variety of geometric judgments. Adaptation experiments with inverting glasses (Stratton, 1897; Kohler, 1962) provide a good illustration why one needs to examine different variables: In these experiments, subjects were fitted with inverting glasses making the stimuli appear “upside down.” First, subjects had difficulty in operating with these glasses but with practice they became quite adept in complex behaviors, such as riding a bicycle in city streets. However, in general, subjects did not experience the stimulus flipping to its normal, instead of upside-down appearance. Hence, at the *perceptual level*, the system did not compensate for and correct the effect of inverting glasses but it did so at the *sensorimotor level* (Harris, 1965; Linden et al., 1999). Hence, careful definitions of dependent and independent variables to reflect the multi-faceted aspects of our brain processes and behaviors are important to explore 4D performance. Random dot stereograms have been used to generate 3D phenomenal experience from 2D images without any information other than disparity. An adaptation of this approach for 4D percepts may help generate phenomenological 4D experience. How this 4D perception would be is difficult to answer; however, we can extrapolate our experiences that arise when we perceive 3D structures in random dot stereograms or the experience in a 3D movie theater with and without polarizing glasses.

## Outstanding Problems: Generation of 4D Stimuli in a 3D World

Methods for presenting four and higher dimensional stimuli and environments have been developed (D’Zmura et al., 2000) and a 4D maze game is available^2^. As mentioned in the review of previous work, a variety of geometric approaches have been used: The projection, slicing, and rotation methods. Some studies provided additional cues to enhance 4D representations. New technologies, such as virtual and augmented reality, can create complex representations of 4D, however, the broader question is rather theoretical: Human retina has a 2D structure onto which the environment needs to be projected. In the case of a 3D environment, the optics of the eye and the physical properties of our 3D environment dictate the use of projective geometry to describe how the distal stimulus is transformed into the corresponding proximal stimulus. How can a 4D world be projected onto 2D proximal stimulus? First, even though we cannot physically build 4D objects, we have mathematical languages to describe and reason about arbitrary spatial dimensions. In fact, some of the early evidence about the possibility of 4D representations came from some mathematicians’ phenomenal reports of 4D perceptions during their studies of 4D spaces (Davis et al., 1995). But unlike 3D to 2D projections, where physical properties of the environment and the physiology of the eyes determine a *specific type of projection*, there is *a priori* no such constraint for 4D. There are infinitely many ways of projecting a higher dimension to a lower one, and without any constraints, which one is most appropriate, remains an open question.

## Outstanding Problems: Practice or No Practice, Feedback or No Feedback

Perceptual learning is a complex process that depends on multiple factors such as motivation, attention, feedback, and amount of practice. Given the drastic changes it implies, learning 4D representations is likely to necessitate time for adaptation. However, how much time this adaptation will take is not known. Providing feedback can help accelerate this process. However, a major issue with feedback is that subjects can, consciously or unconsciously, discover low-level cues that can lead to successful responses without necessarily building 4D representations. This observation points to another fundamental problem, viz., how to eliminate stimulus artifacts that correlate positively with the task.

## Outstanding Problems: Elimination of Artifacts

A major problem in studying 4D is to determine whether subjects’ behavioral success can be based on cues (as mentioned above, some studies used additional cues such as color or principal vanishing points) and factors that are not inherently 4D. For example, Aflalo and Graziano (2008) used two theoretical models of path integration that were designed to solve the puzzle by using *only* 3D spatial reasoning and looked for better performance than these models to infer 4D capabilities. However, Wang (2014a) noted that path integration can be solved algebraically without building 4D representations. Frank (2014) and Wang (2014b) argued whether hypervolume estimation requires 4D representations. Selection of experimental variables and the design of the experiments require a careful analysis of all artifacts and alternative strategies without 4D representations in order to conclude that indeed humans can build 4D representations.

## Outstanding Problems: Inter-Subject Variability

Another issue that needs further investigation is inter-subject variability, as exemplified by the discussion of Miwa et al.’s (2018) results above. It is conceivable that, because of factors such as attention and motivation, some subjects may take longer or may need different training strategies to build 4D representations. Given that even simple visual illusions are not universally experienced (Phillips, 2019), there is also the possibility that only a subset of humans can achieve this competence. Subject populations in these studies were relatively small and composed mainly of college students. It may be also possible that humans lose their ability of building high-dimensional spaces early in childhood as observed in various “critical periods” in development. Hence, there is a need to further analyze inter-subject variability in order to be able to generalize the results.

## What Can Machine Learning Tell Us About 4D Representations?

It has been shown that 3-layer neural networks are “universal approximators,” in that they can learn arbitrary input-output mappings with arbitrary precision (Funahashi, 1989; Hornik et al., 1989, 1990). However, the success of these networks in solving practical problems has been severely constrained in the past by limited training data sets and limited computational power to carry out the training. Recently “deep neural networks” have shown success beyond the performance levels obtained by traditional computer vision and pattern recognition algorithms. In addition to rich data sets and enhanced computing power, this success can be traced to the fact that several layers of these networks include *a priori* computations (e.g., convolutions) that are aimed to capture some of the desired output properties, such as position invariance. By the “*no-free lunch*” (Wolpert, 1996) and “*bias-variance*” (Geman et al., 1992) theorems, it is clear that introducing such *a priori* biases to networks can limit their domain of universality while enhancing their performance in applications in which the bias matches a desired output property, e.g., position invariance. Hence one way to study how 4D representations can be built is through training a variety of neural network architectures, each including different *a priori* structures that reflect *a priori* biases, to find networks that can and those that cannot learn 4D representations.

## Neuroimaging Techniques Pertinent to the Presence or Absence of Solid 4D Percepts

### Decoding 4D Percepts From fMRI Signals

As noted above, improvements in task performance in a 4D environment alone may not be sufficient to conclude that humans can build 4D representations due to potential artifact by factors that are not inherently 4D. This problem persists when one is to test 4D representations in the brain by using neuroimaging techniques such as functional magnetic resonance imaging (fMRI). Studies for 3D perception have used electrophysiological techniques (Ohzawa et al., 1990; DeAngelis et al., 1991; Tsao et al., 2003) and fMRI (Tsao et al., 2003; Preston et al., 2008; Orban, 2011) to identify brain areas that represent 3D information. These studies reported that 3D information can be extracted from activations in multiple visual areas along with the hierarchy of the visual system. In particular, it has been shown that a particular visual area such as V3B/KO is involved in integration of disparity and motion cues to depth (Ban et al., 2012). More recently, the entorhinal cortex has been shown to be involved in representation of 3D space (Kim and Maguire, 2019). Based on these findings in studies for 3D perception, it can be hypothesized that visual areas and the entorhinal cortex come to be involved in representation and integration of 4D information once human subjects acquire 4D percept. More concretely, if activations in these areas accurately predict subjects’ perceptual report and/or performance in 4D visual tasks, it will strongly support that subjects actually perceive an object in 4D space.

## Conclusion

Perceiving higher spatial dimensions beyond 3D has been a longstanding quest for a broad range of researchers including but not limited to philosophers, mathematicians, psychologists, neuroscientists, developers of virtual reality, and engineers. A success in this endeavor will literally create a new dimension for the human mind. It may be possible to reach not only 4 but even higher dimensions. If such an ability exists, its training and testing pose formidable challenges. In this article, we reviewed uncertainties pertaining to whether observers truly learn and represent a 4D scene or rather rely on indirect lower dimensional cues to perform a task which is within a 4D visual space. This high-risk high-return topic has the potential to completely transform our understanding of brain representations and their plasticity.

## Author Contributions

HO and AY drafted the work with indeed more contribution of HO. AY selected and organized the figure. KS provided the draft of the imaging part.

## Conflict of Interest

The authors declare that the research was conducted in the absence of any commercial or financial relationships that could be construed as a potential conflict of interest.

## Footnotes

1 With the exception of those lines that are parallel to the projection plane; these lines remain parallel under perspective projection.

2 http://www.urticator.net/maze/
